# Improved Scheduling Mechanisms for Synchronous Information and Energy Transmission

**DOI:** 10.3390/s17061343

**Published:** 2017-06-09

**Authors:** Danyang Qin, Songxiang Yang, Yan Zhang, Jingya Ma, Qun Ding

**Affiliations:** Key Laboratory of Electronics Engineering, College of Heilongjiang University, Harbin 150080, China; songxiang_yang@foxmail.com or 2151299@s.hlju.edu.cn (S.Y.); 2161362@s.hlju.edu.cn (Y.Z.); 2161365@s.hlju.edu.cn (J.M.); qunding@yahoo.cn or 1984008@hlju.edu.cn (Q.D.)

**Keywords:** wireless sensor networks, synchronous information and energy transmission, ergodic channel capacity, scheduling mechanism

## Abstract

Wireless energy collecting technology can effectively reduce the network time overhead and prolong the wireless sensor network (WSN) lifetime. However, the traditional energy collecting technology cannot achieve the balance between ergodic channel capacity and average collected energy. In order to solve the problem of the network transmission efficiency and the limited energy of wireless devices, three improved scheduling mechanisms are proposed: improved signal noise ratio (SNR) scheduling mechanism (IS2M), improved N-SNR scheduling mechanism (INS2M) and an improved Equal Throughput scheduling mechanism (IETSM) for different channel conditions to improve the whole network performance. Meanwhile, the average collected energy of single users and the ergodic channel capacity of three scheduling mechanisms can be obtained through the order statistical theory in Rayleig, Ricean, Nakagami-*m* and Weibull fading channels. It is concluded that the proposed scheduling mechanisms can achieve better balance between energy collection and data transmission, so as to provide a new solution to realize synchronous information and energy transmission for WSNs.

## 1. Introduction

In recent years, battery-powered wireless communication devices have been developed rapidly, and the methods of prolonging the network lifetime by collected energy have attracted much interest from researchers. Wireless energy collecting (EC) is particularly important for wireless sensor networks (WSNs) because of the limited energy supply [1,2]. It is quite difficult and expensive to replace or charge the battery for the devices, especially in some special circumstances, so a sustainable energy supply is demanded. The common sources of sustainable energy, however, are highly dependent on weather conditions, such as solar and wind energy. It is a practical method to collect the energy from the surrounding environment or to transmit energy for low-power devices by radio frequency (RF).

RF signals can transmit both message and energy so it is possible to combine the message delivery and energy collection as a complete transmission system. The limitations of practical circuit, however, make the receivers hardly achieve energy collection and information decoding by the same signal. Therefore, message-energy time division transmission is a reasonable strategy to realize synchronous wireless information and power transmission (SWIPT), the performance of which will greatly depend on the scheduling mechanism. The balance between message and energy transmission scheduling is illustrated by the boundary of rate-energy (R-E) domain so as to transform the balance issue into a boundary model [3]. In [4,5], the receiver assumes that energy can be collected from the signal used to carry out the message decoding (MD), while the existing technology that is limited by practical circuit cannot achieve this assumption. Thus, the researchers proposed two kinds of realistic reception mechanisms in [6], in which the receiver could switch between EC and MD, or could divide the receiving signal into the MD part and the EC part.

Currently, multi-user systems based on SWIPT are being studied to meet the requirements of real energy-limited networks, such as WSNs. SWIPT systems, designed for broadcast channels and interference channels, are described in [7,8], respectively, and the best transport protocols are also presented. The multi-user multi-input single-output SWIPT system is introduced in [9] with a newly proposed beamforming optimization design, which can maximize the energy collection of EC receiver under the constraints of signal-interference-plus-noise-rate. The multi-user time division multiple access (TDMA) system in [10,11] will transmit the energy and data in the uplink channel and the downlink channel, respectively, with an optimal uplink and downlink time distribution mechanism presented to maximize the total transmission throughput or the equal transmission throughput in the same duration time.

For a message-only transmission system, a multi-user scheduling mechanism is usually designed to adopt the independent and time-varying multipath fading characteristics so as to establish the multi-user diversity [12,13]. With this method, the user can transmit and receive messages in a good channel environment throughout the time slots. For example, in the maximum throughput scheduling mechanism, the user with the highest SNR will be selected to transmit data, so as to obtain the maximum system capacity. However, the users with poor channel conditions cannot access the channel at all. In order to prevent the unfairness, the normalized SNR (N-SNR) scheduling mechanism is proposed to maximize the user’s normalized SNR, thus to improve the user’s system capacity greatly. Another consideration to realize the balance between system capacity and channel quality is to maintain the equal throughput [14,15], which can be achieved by keeping the average traffic of all users at the lowest level in each slot. The multi-user scheduling mechanism can expand the multi-user diversity (MUD) and maintain the system capacity and channel quality in a balanced state for a long time, but this mechanism has not yet been achieved in SWIPT [16,17,18,19]. In this paper, three improved scheduling mechanisms are presented to realize multi-user scheduling in a SWIPT system, which can achieve a better balance between data transmission and energy collection during the active state for WSNs. The main contributions of this paper can be summarized as follows:

(1) The improved SNR and N-SNR scheduling mechanisms for multi-user SWIPT system are proposed, wherein the users are sorted in ascending order according to SNR and N-SNR, respectively. The *j*th user, who has the *j*th smallest SNR or N-SNR, will be selected to deliver the data, and the rest users will collect the energy from surroundings. Obviously, a lower *j* means that the user selected for data transmission has a poorer channel condition. Meanwhile, the users with better channel conditions will perform the energy collection. Thus, the lower *j* will cause a smaller ergodic capacity and a larger average collected energy (ACE).
(2) The improved ET scheduling mechanism is designed, wherein the users are sorted in ascending order according to N-SNR. $S_{a}$ is a specific set of ordinal. The user, who has an N-SNR order in the set of $S_{a}$ and has the minimum average throughput, will be selected to transmit data in each time slot. Therefore, a smaller set of $S_{a}$ will cause a larger ACE and a smaller ergodic capacity. In addition, this paper gives the necessary conditions for the user to achieve ET.
(3) The improved SNR, N-SNR and ET scheduling mechanisms are analyzed using order statistical theory for heterogeneous (independent or non-identically distributed) Nakagami-*m*, Weibull, Ricean and Rayleigh fading channels. In addition, three types of scheduling mechanisms and the relative approximate expressions are established for ACE and ergodic channel capacity (ECC) of a single user in corresponding fading models.

## 2. Model Introduction

This section will describe the SWIPT system model, the channel model, and the EC model adopted in this paper.

### 2.1. Symbols and Formulas

Some symbols and functions used in this paper should be explained first to make the discussion clear. Γ(s,x) is γ function without supremum, which is regarded as $\Gamma \left(s,x\right)=\int_{x}^{\infty }t^{s-1}e^{-t}$d*t*. The γ function Γ(s) is regarded as $\Gamma \left(s\right)=\int_{0}^{\infty }t^{s-1}e^{-t}$d*t* when *s* is a positive non-integer, and Γ(s)=(s−1)! when *s* is a positive integer. *E*[·] indicates the expected value. $I_{0}\left(\cdot \right)$ is the first kind modified Bessel function with order zero, which is regarded as $I_{0}\left(x\right)=J_{0}\left(ix\right)$. $J_{0}\left(x\right)$ is the first kind Bessel function with order zero, which is regarded as $J_{0}\left(x\right)=\Sigma_{m=0}^{\infty }\frac{{\left(-1\right)}^{m}}{m!\Gamma (m+1)}{\left(\frac{x}{2}\right)}^{2m}$. $Q_{1}\left(a,b\right)$ is first order Marcum *Q* function, which is regarded as $Q_{1}\left(a,b\right)=\int_{b}^{\infty }xe^{-\frac{\left(x^{2}+a^{2}\right)}{2}}I_{0}\left(ax\right)$d*x*. $G_{p,q}^{m,n}\left[z|\begin{array}{c} a_{1},...,a_{p}\\ b_{1},...,b_{q} \end{array}\right]=\frac{1}{2\pi i}\int_{L}g\left(s\right)z^{-s}ds$ is the Meijer G function [20]. $E_{1}\left(x\right)=\int_{1}^{\infty }\frac{e^{-tx}}{t}$d*x* is the first order exponential integral function. $\Psi \left(x\right)=\frac{d}{dx}\ln\left(\Gamma \left(x\right)\right)=\frac{\Gamma^{\prime }\left(x\right)}{\Gamma (x)}$ is the digamma function. C=0.5772156649...=−Ψ(1) is the Euler’s constant [21]. ${}_{p}F_{q}\left(\begin{array}{c} a_{1},...,a_{p}\\ b_{1},...,b_{q} \end{array}\right)=\Sigma_{n=0}^{\infty }\frac{{\left(a_{1}\right)}_{n}...{\left(a_{p}\right)}_{n}}{{\left(b_{1}\right)}_{n}...{\left(b_{q}\right)}_{n}}\frac{z^{n}}{n!}$ is a generalized hypergeometric distribution function [21].

### 2.2. System Model

**Figure 1:**
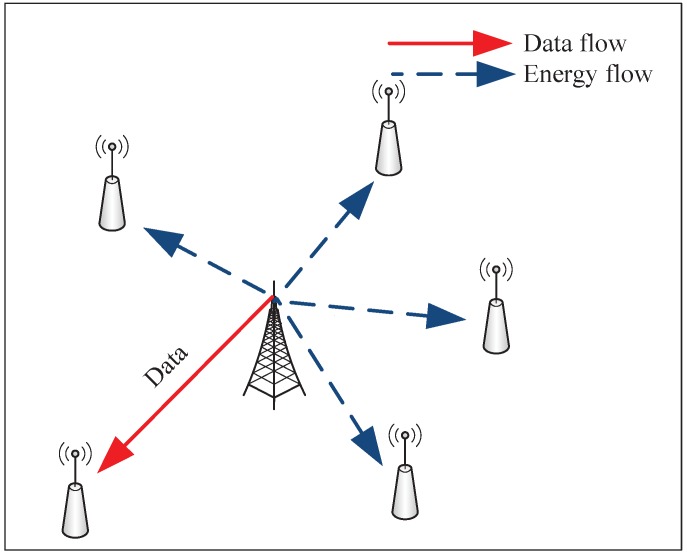
Multiuser SWIPT system.

A SWIPT system is modeled with one network access port (NAP) supplied by continuous energy and *N* battery-powered subscriber terminals (STs) in this section, which is used to research the downlink transmission. Assume that the NAP will always transmit data to each ST with the dedicated packets. STs will adopt time-slot controller in the receivers to perform data encoding or energy collection. Figure 1 shows that the NAP selects a user to transmit the data, and the remaining free users collect energy from the received signal in a certain time slot. In time slot *t*, the NAP will send the data to the selected user, namely user *n*. Then, the received signal will be:

(1) $$\begin{array}{c} r_{n}=\sqrt{Ph_{n}}e^{j\theta_{n}}x+z_{n},\forall n\in \left\{1,...,N\right\}, \end{array}$$

where *P* is the transmission power of the NAP, and *x* is a baseband signal with the average power normalized to 1, namely $E[|x{|}^{2}]=1$. $\theta_{n}$ and $\sqrt{h_{n}}$ represent the phase and amplitude of the channel fading coefficients, respectively. $z_{n}$ denotes a Gaussian white noise with zero mean and variance $\sigma^{2}$. $r_{n}$ is the data throughput from NAP to user *n*, which is an important parameter to calculate the average ergodic capacity and to select the user to deliver data in the improved ET scheduling mechanism.

### 2.3. Energy Collecting Model

**Figure 2:**
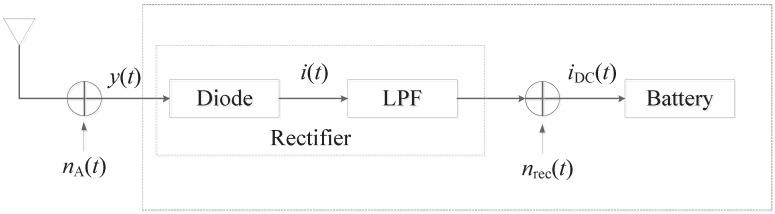
Energy collecting model.

The energy collecting model of Zhou [21] is adopted as the basic framework as shown in Figure 2, in which y(t) is the received RF band signal; $i_{DC}\left(t\right)$ means the direct current (DC) signal for battery charging; $n_{A}\left(t\right)$ is a narrow-band Gaussian noise; and $n_{rec}\left(t\right)$ is the additive noise introduced by the rectifier. The collected energy caused by noise is so small as to be ignored in this paper. ACE is given by $EC=\eta E[i_{DC}\left(t\right)]=\eta hp$, where *h* is the channel energy, and η is the conversion efficiency with the value changing from 0 to 1. The remaining parameters are shown in Table 1.

### 2.4. Fading Channel

The channels between the NAP and the STs are block fading. Then, the channel conditions will remain stable in a time slot but may change in next time slot. The fading factors of different user links are assumed to be statistically independent, so it is referred to as Ricean, Nakagami-*m*, Weibull and Rayleigh fading channel models in this paper. The channel energy gain obtained from user *n* is represented by $\Omega_{n}=E\left[h_{n}\right],n=1,...,N$. Table 2 summarizes the probability density functions (PDF) and the cumulative distribution functions (CDF) of channel energy $h_{n}$ for various fading channel models:

(1) Ricean fading channel: The Ricean fading channel is suitable for short distance RF EC situation, where the NAP is within the range of the user’s sight. If $\sqrt{h_{n}}$ complies with the Rician distribution, the channel power gain $h_{n}$ will obey the non-central $\chi^{2}$ distribution. The first order Marcum-Q equation [22] is used to facilitate the performance analysis of different scheduling mechanisms in Ricean fading channels, namely $Q_{1}\left(a,b\right)\approx e^{-e^{\nu (a)}b^{\mu (a)}}$. μ(a) and ν(a) are nonnegative parameters that can decrease the error between the theoretical value and the approximation of the function:
  (2) $$\begin{array}{c} \mu \left(a\right)=\left\{\begin{array}{cc} 2+\frac{9}{8(9\pi^{2}-80)}a^{4}, & a\ll 1,\\ 2.174-0.592a+0.593a^{2}-0.092a^{3}+0.005a^{4}, & \mathrm{otherwise}, \end{array}\right. \end{array}$$
  (3) $$\begin{array}{c} \nu \left(a\right)=\left\{\begin{array}{cc} \frac{45\pi^{2}+72\ln2+36C-496}{64(9\pi^{2}-80)}a^{4}-\frac{a^{2}}{2}-\ln2, & a\ll 1,\\ 0.327a-0.840-0.740a^{2}+0.083a^{3}-0.004a^{4}, & \mathrm{otherwise}. \end{array}\right. \end{array}$$
  The accuracy of the approximation will decrease with *K* (Ricean factor) increasing. Applying an approximation of Marcum-Q equation, the cumulative distribution function of the channel power gain is $F_{h_{n}}\left(x\right)\approx 1-e^{-\beta_{n}x^{\mu^{\prime }}}$, where $\mu^{\prime }=\mu \left(\sqrt{2K}\right)/2$, $\beta_{n}=e^{\nu (\sqrt{2K})}{\left(2\left(K+1\right)\Omega_{n}^{-1}\right)}^{\mu (\sqrt{2K})/2}$. It can be seen that the result of the cumulative distribution function is similar to that of Weibull fading channel.
(2) Weibull fading channel: Weibull fading channel is suitable for broadcast channels in narrowband body area networks. The network embedded in the human body can only be powered by wireless energy transmission. If the channel parameter $\sqrt{h_{n}}$ of sensor *n* is in accordance with the Weibull distribution, the channel power gain $h_{n}$ will follow the Weibull distribution, $\forall n\in \left\{1,...,N\right\}$.
(3) Nakagami-*m* fading channel: Nakagami-*m* fading channel can cope with the indoor wireless energy collecting model. The fading channels can be distinguished by the shape parameter *m*, which is the index of the channel fading degree. The smaller the *m* is, the quicker the channel fading will be. *m* is considered as the parameter for auxiliary analysis. The Nakagami-*m* fading channel energy obtained by the users will follow the Erlang distribution.
(4) Rayleigh fading channel: Rayleigh fading channel model is a particular case of the above channel model. If $\sqrt{h_{n}}$ of user *n* conforms to Rayleigh distribution, the channel power gain will obey an exponentially distributed.

### 2.5. Reference Scheduling Mechanism

Two well-known scheduling mechanisms, round robin (RR) and the traditional equal throughout (ET), will be discussed in this part, to serve as a reference scheme. In order to facilitate the comparison, each user’s ECC and ACE in the reference scheduling mechanisms will be analyzed:

(1) RR mechanism: RR scheduling strategy confers the channel to the users orderly, and the NAP does not need to know the channel gain of the different users. Therefore, the probability of each user receiving messages is $\frac{1}{N}$, so the probability of collecting energy is $1-\frac{1}{N}$. The ergodic capacity $E(C_{U_{n}})$ achieved by user *n* (denoted by $U_{n}$) is $\frac{1}{N}$ times of $E(C_{U_{n,f}})$:
  (4) $$\begin{array}{c} E\left(C_{U_{n}}\right){|}_{RR}=\frac{1}{N}E\left(C_{U_{n,f}}\right), \end{array}$$
  where $E\left(C_{U_{n,f}}\right){|}_{RR}=\int_{0}^{\infty }\log_{2}\left(1+\bar{\gamma }x\right)f_{h_{n}}\left(x\right)dx$, $\bar{\gamma }≜\frac{P}{\sigma^{2}}$, are derived in [23] and summarized in Table 3. ACE of user *n* is $E\left[{EC}_{U_{n}}\right]=\left(1-\frac{1}{N}\right)\eta P\Omega_{n}$.
(2) ET Mechanism: The long-term average traffic of all users will be balanced by scheduling users with minimum moving average traffic in each time slot according to the traditional ET scheduling. In time slot *t*, the user $n^{*}$ for transmitting messages should satisfy
  (5) $$\begin{array}{c} n^{*}=\arg\min r_{n}\left(t-1\right),n\in \left\{1,...,N\right\}. \end{array}$$

The definition in Table 3 is taken for Weibull fading, namely $\lambda_{n}^{\prime }=\frac{\Gamma (1+\frac{1}{k})}{\bar{\gamma_{n}}}$, and the parameters *a* and *b* are the smallest positive integer to satisfy $\frac{b}{a}=k$. The function Δ(x,y) is regarded as $\Delta \left(x,y\right)=\frac{y}{x},\frac{y+1}{x},...,\frac{y+x-1}{x}$ [24]. $r_{n}\left(t-1\right)$ represents the average traffic of user *n* from the previous time slot to time slot t−1 as in Equation (6):

(6) $$\begin{array}{c} r_{n}\left(t\right)=\left\{\begin{array}{cc} \left(1-\beta \right)r_{n}\left(t-1\right)+\beta C_{n}\left(t\right), & \mathrm{if}\;\mathrm{user}\;n\;\mathrm{is}\;\mathrm{selected},\\ \left(1-\beta \right)r_{n}\left(t-1\right), & \mathrm{otherwise}, \end{array}\right. \end{array}$$

where $C_{n}\left(t\right)$ is the reachable information rate of user *n* in time slot *t* and will satisfy $C_{n}\left(t\right)=\log_{2}\left(1+\bar{\gamma }h_{n}\left(t\right)\right)$; $h_{n}\left(t\right)$ is the channel power gain; β∈(0,1) is a decreasing smooth factor (for example, $\beta =\frac{1}{t}$) to guarantee the average transmission capacity $r_{n}\left(t\right)$ converging to the overall average $E[C_{U_{n}}]$ for the fixed fading process $h_{n}\left(t\right)$ [25]. It is noted that the traditional ET scheduling strategy only requires the NAP to know the instantaneous channel of the selected user. In the traditional ET scheduling mechanism, the average capacity of user *n* should satisfy Equation (7):

(7) $$\begin{array}{c} E\left[C_{U_{n}}\right]=p_{n}E\left[C_{U_{n,f}}\right]=r,\forall n\in \left\{1,...,N\right\}. \end{array}$$

The average capacity is forcefully set to *r*, which is the equal transmission capacity achieved by all users in the network. In Equation (7), $p_{n}$ is the channel access probability (CAP) of user *n*, and $E[C_{U_{n,f}}]$ is the average full-time access capacity of user *n*, which is described detailedly in Table 2. Therefore, in order to obtain the transmission capacity *r*, the CAP of user *n* should be $p_{n}=\frac{r}{E[C_{U_{n,f}}]}$. Since there is $\sum_{n=1}^{N}p_{n}=1$, the capacity expectation of ET scheduling mechanism will be as Equation (8):

(8) $$\begin{array}{c} E\left[C_{U_{n}}\right]{|}_{ET}={\left(\sum_{n=1}^{N}\frac{1}{E[C_{U_{n,f}}]}\right)}^{-1}. \end{array}$$

Furthermore, the scheduling probability required by user *n* is reduced to $p_{n}={\left(\sum_{i=1}^{N}\frac{E[C_{U_{n,f}}]}{E[C_{U_{i,f}}]}\right)}^{-1}$, and the relative ACE collected by the same user is $E\left[EC_{U_{n}}\right]=\left(1-p_{n}\right)\eta P\Omega_{n}$.

In a SWIPT system, RR and traditional ET scheduling cannot achieve the balance between ECC and ACE. Therefore, three scheduling mechanisms for different channel conditions are proposed to solve such problem.

## 3. Improved SNR Scheduling Mechanism

In this part, an improved SNR scheduling mechanism (IS2M) is proposed to allow the user with the *j*th$\left(j\in \left\{1,...,N\right\}\right)$ order to receive messages.

### 3.1. IS2M

The fact that all users have the same *P* and $\sigma^{2}$ makes it possible to replace the SNR order by the channel power gain to realize user selection. Thus, the user transmitting messages should satisfy $n^{*}=\arg orderh_{n},n\in \left\{1,...,N\right\}$, where argorder is define as the *j*th ascending order.

### 3.2. Performance Analysis

In order to study ECC and ACE of a single user in IS2M, the instantaneous channel power gain $h_{n}$ of all users will satisfy the ascending order $h_{\left(1\right)}\leq h_{\left(2\right)}\leq ...\leq h_{\left(N\right)}$, where $h_{\left(j\right)}$ is the *j*th minimum channel power gain. The probability density function of $h_{\left(j\right)}$ in independent or non-identical distribution channel is as follows:

(9) $$\begin{array}{c} f_{h_{\left(j\right)}}\left(x\right)=\sum_{n=1}^{N}\sum_{P_{n}}\prod_{l=1}^{j-1}F_{h_{i_{l}}}\left(x\right)f_{h_{n}}\left(x\right)\prod_{l=j}^{N-1}\left(1-F_{h_{i_{l}}}\left(x\right)\right). \end{array}$$

$f_{h_{n}}\left(x\right)$ and $F_{h_{n}}\left(x\right)$ in Equation (9) are probability density function and the cumulative distribution function of the channel power gain of user *n* in different fading models, respectively. For the user with a given order *j*, its ECC will be:

(10) $$\begin{array}{c} \begin{array}{cc} E[C_{j,U_{n}}]= & \int_{0}^{\infty }\log_{2}\left(1+\bar{\gamma }x\right)f_{h_{n}}\left(x\right)\sum_{P_{n}}\prod_{l=1}^{j-1}F_{h_{i_{l}}}\left(x\right)\prod_{l=j}^{N-1}\left(1-F_{h_{i_{l}}}\left(x\right)\right)dx. \end{array} \end{array}$$

The average total collected energy of user *n* will be:

(11) $$\begin{array}{c} \begin{array}{c} E\left[EC_{j,U_{n}}\right]=\eta P\int_{0}^{\infty }xf_{h_{n}}\left(x\right)\left(1-\sum_{P_{n}}\prod_{l-1}^{j-1}F_{h_{i_{l}}}\left(x\right)\prod_{l=j}^{N-1}\left(1-F_{h_{i_{l}}}\left(x\right)\right)\right)dx. \end{array} \end{array}$$

Table 4 shows the limits of ECC in different fading channels. For Rician fading channel, the capacity approximation obtained by the approximate value of the Marcum-Q function cannot be regarded as the lower bound. $U_{n,r}$ is regarded as $U_{n,r}=\left\{\left(u_{1},...,u_{N-j+r}\right)|\left(u_{1},...u_{r}\right)=\left(c_{1},...,c_{r}\right),\left(u_{r+1},...,u_{N-j+r}\right)\;=\left(i_{j},...,i_{N-1}\right)\right\}$, $\forall \left(i_{1},...,i_{N-1}\right)\in P_{n}$, $\forall \left(c_{1},...c_{r}\right)\subset \left(i_{1},...,i_{j-1}\right)$. $S_{m,r}$ is regarded as $S_{m,r}=\left\{\left(s_{1},...,s_{N-j+r}\right)|s_{t}\in \left\{0,...,m-1\right\}\right\}$, $\forall t\in \left\{1,...,N-j+r\right\}$. Table 5 shows the ACE of a single user in different fading channels.

## 4. Improved N-SNR Scheduling Mechanism

Since IS2M may limit some users to receive information when they are in different channel environments, an improved N-SNR scheduling mechanism is presented by selecting users with the *j*th ascending N-SNR order to transmit messages.

### 4.1. INS2M

The user transmitting messages based on INS2M should satisfy $n^{*}=\arg order\frac{h_{n}}{\Omega_{n}},n\in \left\{1,...,N\right\}$. The normalization in the above equation will ensure that the average channel visiting times for all users are almost the same, so that the proportional fairness between ECC and ACE can be guaranteed at some degree.

### 4.2. Performance Analysis

$X_{n}=h_{n}/\Omega_{n}\left(\forall n\in \left\{1,...,N\right\}\right)$ follows the same distribution with $h_{n}$, but possesses the meaning of units. Assuming that all the users have the same shape parameters, and their probability density functions and cumulative distribution functions are defined by $f_{h_{n}}\left(x\right)$ and $F_{h_{n}}\left(x\right)$ in Table 2, where there is $\Omega_{n}=1$. In the following part, ECC and ACE of a single user will be discussed based on INS2M. The random variable of the *j*th user is represented by $X_{j}$, and the relative probability density function can be expressed as:

(12) $$\begin{array}{c} \begin{array}{cc} f_{X_{j}}\left(x\right)= & N\left(\begin{array}{c} N-1\\ j-1 \end{array}\right)f_{X}\left(x\right){\left[F_{X}\left(x\right)\right]}^{j-1}{\left[1-F_{X}\left(x\right)\right]}^{N-j}. \end{array} \end{array}$$

Therefore, ECC of user *n* is:

(13) $$\begin{array}{c} E\left[C_{j,U_{n}}\right]=\frac{1}{N}\int_{0}^{\infty }\log_{2}\left(1+{\bar{\gamma }}_{n}x\right)f_{X_{\left(j\right)}}\left(x\right)dx. \end{array}$$

${\bar{\gamma }}_{n}=\bar{\gamma }\Omega_{n}$ is the mean of SNR. ECC is normalized by *N*, the reciprocal of which denotes the possibility of a normalized channel to be selected as the data transmission path, since the normalized channel is independently and identically distributed.

ACE of user *n* is:

(14) $$\begin{array}{c} \begin{array}{cc} E[EC_{j,U_{n}}] & =\eta P\Omega_{n}\int_{0}^{\infty }x\left(f_{X}\left(x\right)-\frac{1}{N}f_{X_{j}}\left(x\right)\right)dx=\eta P\Omega_{n}\left[1-\frac{E\left[X_{\left(j\right)}\right]}{N}\right]. \end{array} \end{array}$$

Table 6 and Table 7 show ECC and ACE expectations of a single user based on INS2M in different independent identically distributed fading channels with high SNR. In these two tables, the set $L_{m,l}$ is defined as $L_{m,l}=\left\{\left(i_{0},...,i_{m-1}\right)|i_{s}\in \left\{0,...,l\right\},\forall s\in \left\{0,...,m-1\right\},and\sum_{m-1}^{s=0}i_{s}=l\right\}$.

## 5. Improved Equal Throughput Scheduling Mechanism

An improved equal throughput scheduling algorithm is proposed to balance the whole network transmission load among all users equally, and to adjust the information transmission according to ACE of the users.

### 5.1. IETSM

A set of $S_{a}$ is defined in the IETSM. The user with the minimum information transmission throughput will be selected, whose N-SNR order *j* should belong to $S_{a}$. The algorithm normalizes the instantaneous SNR by the average of each user in each time slot. While other users will collect the energy from the received signals. It should be noted that the IETSM may not be feasible in all cases, which depends on $S_{a}$ and the user’s average channel power. For the cases of IETSM being feasible, the orders in $S_{a}$ could control the balance between the information throughput and the energy collection. $O_{U_{n}}$ represents the N-SNR order of user *n*, and $O_{U_{n}}\in \left\{1,...,N\right\}$. IETSM will select user $n^{*}$ to transmit messages in time slot *t*, which satisfies $n^{*}=\underset{O_{U_{n}}\in S_{a}}{\arg\min}r_{n}\left(t-1\right)$. $r_{n}\left(t-1\right)$ is the average throughput of user *n* in the preceding t−1 slot, then the transmission state of the user will be updated asynchronously.

### 5.2. Performance Analysis

The feasibility of ET and ACE of a single user in the IETSM will be analyzed in this part. The average ECC of user *n* is:

(15) $$\begin{array}{c} E\left[C_{U_{n}}\right]=E\left[C_{U_{n}}|O_{U_{n}}\in S_{a}\right]\times \mathrm{Pr}\left(O_{U_{n}}\in S_{a}\right). \end{array}$$

Let $\mathrm{Pr}\left(O_{U_{n}}\in S_{a}\right)=\left|S_{a}\right|/N$, and then the average ECC decreases to:

(16) $$\begin{array}{c} \begin{array}{cc} E[C_{U_{n}}] & =\frac{|S_{a}|}{N}\int_{0}^{\infty }\log_{2}\left(1+\bar{\gamma_{n}}x\right)\left(\frac{1}{|S_{a}|}\sum_{j\in S_{a}}f_{X_{j}}\left(x\right)\right)dx\times \mathrm{Pr}\left(U_{n}|O_{U_{n}}\in S_{a}\right)\\ & =\sum_{j\in S_{a}}E\left[C_{j,U_{n}}\right]{|}_{N-SNR}\mathrm{Pr}\left(U_{n}|O_{U_{n}}\in S_{a}\right), \end{array} \end{array}$$

where $\frac{1}{|S_{a}|}f_{X_{j}}\left(x\right)$ is the likelihood function of the normalized channel with order *j*, and the probability of the user *n* transmitting the messages is $\mathrm{Pr}\left(U_{n}|O_{U_{n}}\in S_{a}\right)=\mathrm{Pr}\left(n^{*}=n|O_{U_{n}}\in S_{a}\right)$. $E\left[C_{j,U_{n}}\right]{|}_{N-SNR}$ is the average ECC that can be realized by user *n* based on INS2M.

To obtain the average ECC, a parameter $p_{n}$ is defined to represent the probability of user *n* being selected with the expression as Equation (17):

(17) $$\begin{array}{c} \begin{array}{cc} p_{n} & ≜\mathrm{Pr}\left(U_{n}\right)=\mathrm{Pr}\left(U_{n}|O_{U_{n}}\in S_{a}\right)\mathrm{Pr}\left(O_{U_{n}}\in S_{a}\right)=\mathrm{Pr}\left(U_{n}|O_{U_{n}}\in S_{a}\right)\frac{|S_{a}|}{N}. \end{array} \end{array}$$

Therefore, the average ECC of user *n* is reduced as Equation (18):

(18) $$\begin{array}{c} \begin{array}{cc} E[C_{U_{n}}]= & \frac{N}{|S_{a}|}\sum_{j\in S_{a}}E\left[C_{j,U_{n}}\right]{|}_{N-SNR}p_{n}=r,\forall n\in \left\{1,...,N\right\}. \end{array} \end{array}$$

The average capacity of all users is forcefully set to *r*. Therefore, the probability of user *n* accessing the channel will be $p_{n}=r/\left(\frac{N}{|S_{a}|}\sum_{j\in S_{a}}E\left[C_{j,U_{n}}\right]{|}_{N-SNR}\right)$. Since there is $\Sigma_{n=1}^{N}p_{n}=1$ all the time, the equal information throughput *r* will be:

(19) $$\begin{array}{c} r=\frac{1}{\frac{1}{N}\sum_{n=1}^{N}\frac{1}{\frac{1}{|S_{a}|}\sum_{j\in S_{a}}E\left[C_{j,U_{n}}\right]{|}_{N-SNR}}}. \end{array}$$

For IETSM, the possibility of all users achieving equal transmission is related to the harmonic average of the mean value of the users’ ECC $E\left[C_{j,U_{n}}\right]{|}_{N-SNR},j\in S_{a}$. It means that the greater order the set of $S_{a}$ contains, the larger the average transmission throughput *r* will be. In addition, the harmonic average implies that the user with poorer channel is more likely to achieve equal transmission. For the fading channels in Table 7, the approximate expression of ECC based on INS2M could be used to obtain the approximate expression of equal transmission as in Equation (19). From Equation (18) and Equation (19), the CAP will be obtained to guarantee user *n* having the same transmission throughput as other active users:

(20) $$\begin{array}{c} p_{n}=\left(\sum_{i=1}^{N}\frac{\sum_{j\in S_{a}}E\left[C_{j,U_{n}}\right]{|}_{N-SNR}}{\sum_{j\in S_{a}}E\left[C_{j,U_{i}}\right]{|}_{N-SNR}}\right),\forall n\in \left\{1,...,N\right\}. \end{array}$$

For the certain combinations of $S_{a}$ and $\Omega_{n}$, the IETSM may not achieve equal transmission for all users. The environmental conditions in Theorem 1 are provided to ensure the feasibility of IETSM.

**Theorem** **1.**  *When* $|S_{a}|>1$, *the IETSM is feasible if and only if the following conditions are satisfied: (1)* $p_{n}\leq \frac{|S_{a}|}{N},\forall n\in \left\{1,...,N\right\}$; *(2)* $\sum_{l=1}^{L}p_{n_{l}}\leq \left[\left(\begin{array}{c} N-1\\ |s_{a}|-1 \end{array}\right)L+\left(\begin{array}{c} L\\ |s_{a}| \end{array}\right)\left(1-|s_{a}|\right)\right]÷\left(\begin{array}{c} N|s_{a}| \end{array}\right),\forall \left(n_{1},...,n_{L}\right)\in C_{L},\forall L=\left|S_{a}\right|,...,N$. $C_{L}$ *in Theorem 1 is the set of all* $\left(\begin{array}{c} NL \end{array}\right)$ *with N and L belong to* $\left\{\right\}n_{1},...,n_{L}\}$ *and* {1,...,N}, *respectively. The second feasible condition can always be satisfied when* $\sum_{n=1}^{N}p_{n}\leq 1$ *and* L=N. *In addition, equal transmission is always feasible when there is* $p_{n}\leq 1$ *for the first condition and* L=N *for the second condition. This scheduling mechanism will be the same to INS2M when* $|S_{a}|=1$.

Next, ACE of a single user will be analyzed. $S_{a}^{C}$ represents the supplementary set of $S_{a}$ with respect to {1,...,N}, then ACE of user *n* is:

(21) $$\begin{array}{c} \begin{array}{cc} E[EC_{U_{n}}]=\; & E\left[EC_{U_{n}}|O_{U_{n}}\in S_{a}^{C}\right]\times Pr\left(O_{U_{n}}\in S_{a}^{C}\right)+E\left[EC_{U_{n}}|O_{U_{n}}\in S_{a}\right]\times Pr\left(O_{U_{n}}\in S_{a}\right)\\ =\; & \eta P\Omega_{n}\left[\int_{0}^{\infty }x\frac{1}{|S_{a}^{C}|}\sum_{j\in S_{a}^{C}}f_{X_{(}j)}\left(x\right)dx\times \frac{|S_{a}^{C}|}{N}+\int_{0}^{\infty }x\frac{1}{|S_{a}|}\sum_{j\in S_{a}}f_{X_{(}j)}\left(x\right)\left(1-\frac{p_{n}N}{|S_{a}|}\right)dx\times \frac{|S_{a}|}{N}\right]\\ =\; & \eta P\Omega_{n}\left[\frac{1}{N}\sum_{j=1}^{N}E\left[X_{j}\right]-\frac{p_{n}}{|S_{a}|}\sum_{j\in S_{a}}E\left[X_{j}\right]\right]=\eta P\Omega_{n}\left[1-\frac{p_{n}}{|S_{a}|}\sum_{j\in S_{a}}E\left[X_{j}\right]\right]. \end{array} \end{array}$$

The users must collect the energy when its N-SNR order lies in the set of $S_{a}^{C}$. Others may collect the energy only when they do not need to transmit messages, whose N-SNR orders belong to $S_{a}$. It can be seen from Equation (17) that $\frac{p_{n}N}{|S_{a}|}$ is the contingent probability of the terminals being selected, and there is $O_{U_{n}}\in S_{a}$. At last, there will be $\sum_{j=1}^{N}X_{j}=\sum_{n-1}^{N}X_{n}$, since $X_{n}$ is a unified term. Thus, there is $\sum_{j=1}^{N}E\left[X_{j}\right]=\sum_{n=1}^{N}E\left[X_{n}\right]=N$.

## 6. Implementation and Performance Analysis

Three scheduling mechanisms are simulated using ISM band with the center frequency as 915 MHz and the bandwidth as 26 MHz. The distance range between transmitter and receiver is from 2 m to 4 m. The packet sending rate is 2 packets per second, and the size of each packet is 16 bits. The additional noise of the receivers has the power of $\sigma^{2}$ = −96 dBm. Using the indoor path loss model [26], the NAP and STs are in the same layer. Assuming that the transmission power of NAP is *P* = 1 W, the antenna gain of NAP and STs are 10 dBi and 2 dBi, respectively. The conversion efficiency is η = 0.5. Now, a system with *N* = 7 is taken into account, and the average channel power gain of a single user is $\Omega_{n}$ = $n\times 10^{-5}$, *n* = 1, ..., 7.

**Figure 3:**
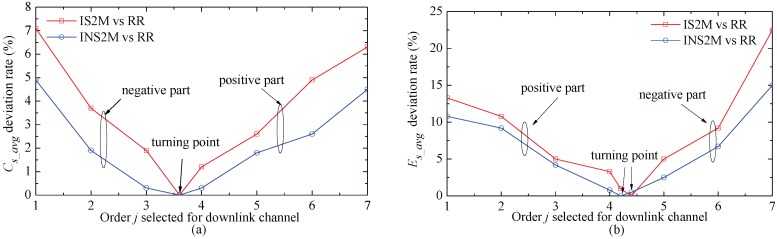
The average system capacity and the total collected energy obtained by IS2M, INS2M and RR scheduling mechanism in the Nakagami-*m* fading channel with N=7 and m=3. (**a**) average system capacity; (**b**) average collected energy.

The performance of the average total information capacity ($C_{s\_avg}$) is calculated by $\Sigma_{n=1}^{N}E\left[C_{j,U_{n}}\right]$, and the average total collected energy ($E_{s\_avg}$) is obtained by $\Sigma_{n=1}^{N}E\left[EC_{j,U_{n}}\right]$. Figure 3 shows the comparisons of $C_{s\_avg}$ and $E_{s\_avg}$ based on IS2M and INS2M with different order *j*s (j=1,...,N). The shape parameters of the independent and identically distributed Nakagami-*m* fading model use the setting of m=3. For INS2M, ACE of the whole system will be increased by 45% when the order *j* declines to 1. Therefore, the order *j* can be used to control the balance between the system capacity and energy collection.

**Figure 4:**
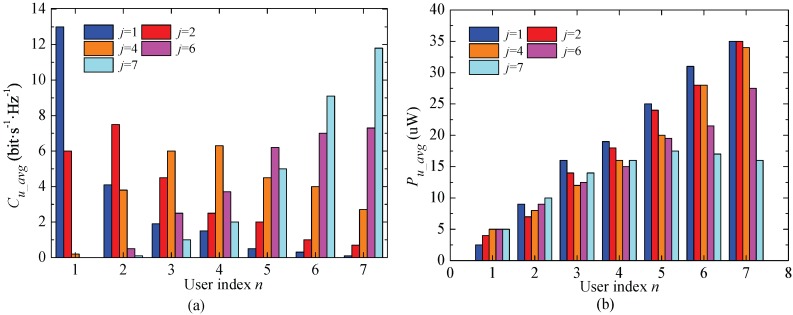
The average system capacity and the total collected energy of single user obtained by IS2M in the Nakagami-*m* fading channel N=7 and m=3. (**a**) ECC of single user; (**b**) ACE of single user.

The collected power is used to replace the energy so as to evaluate the unit average effect, since the collected energy is changing frequently. Figure 4 shows the average user capacity ($C_{u\_avg}$) and the average user collected power ($P_{u\_avg}$) of IS2M with different orders. For any order *j*, IS2M cannot ensure the proportional fairness for all users in different channel environments. The *j*th order user will achieve the highest system capacity, and there is $\Omega_{n}$ = $n\times 10^{-5}$, *n* = 1, ..., 7. Likewise, the amount of collected energy of the users is determined by how often a user is selected to transmit the messages, and how much energy can be collected when some user fails to be selected.

**Figure 5:**
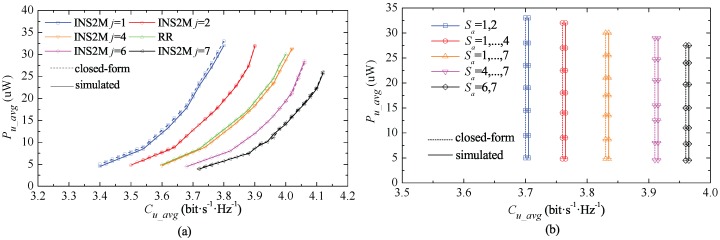
The energy efficiency of INS2M, RR and IETSM in the Ricean fading channel with N=7 and K=6. (**a**) INS2M; (**b**) IETSM.

The performance of INS2M and IETSM over the independent and identically distributed Ricean fading channel with the shape parameter K=6 is shown in Figure 5. It can be seen that the higher the fading factor is, the looser the approximate value is. For instance, when K=18, the differences of ECC and ACE between the theoretical results obtained by approximate expressions and the simulating results are 0.51% and 0.64%, respectively.

In Figure 5a, it can be seen that both INS2M and RR scheduling mechanisms can realize the equal proportional relationship in the aspects of ECC and ACE, since all users are selected fairly in the same time slots. For INS2M, a user could collect more energy when the order *j* decreases from *N* to 1 so as to make ECC decline. For example, the order *j* declining from *N* to 1 will result in an 8% decrease in capacity and a 26% increase in energy collection for the optimal channel users. Therefore, *j* can be chosen according to the requirements of the network.

IETSM can realize the equal transmission for all users as shown in Figure 5b. Thus, equal transmission is feasible for all cases being considered in this study, and all of the conclusions can be verified by Theorem 1. In addition, ACE corresponds to the user’s channel environments. It can be seen that, for the same set of $|S_{a}|$, the lower an order is selected from $S_{a}$, the higher ACE obtained by all users will be, and the lower the whole information capacity will be. Thus, a compromise can be made if $S_{a}$ = {1, 2} and $S_{a}$ = {*N* − 1, *N*} are feasible. In particular, the energy collected by the users in the best and worst channels will increase by 18% and 21.1%, respectively. The transmission capacity will reduce by 6.29%, when $S_{a}$ changes from $S_{a}$ = {6, 7} to $S_{a}$ = {1, 2}.

**Figure 6:**
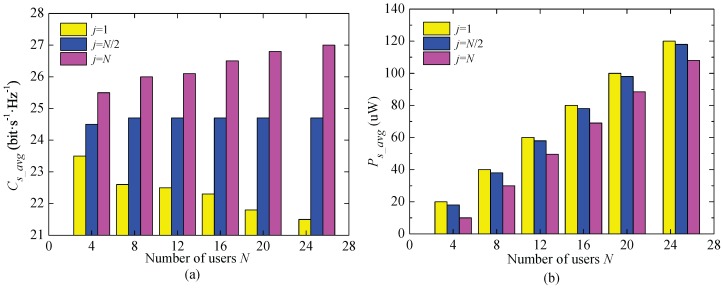
The average system capacity and total collected energy of INS2M with different numbers of users within the independent identically distributed Weibull fading channels, k=1.5. (**a**) the average system capacity; (**b**) the total collected energy.

The impacts of the number of users on ECC and total collected energy achieved by INS2M are shown in Figure 6. In fairness, the average channel power gain of the all users is uniform to $10^{-5}$, namely $\Omega_{n}=\left(\frac{nN}{\sum_{i=1}^{N}i}\right)\times 10^{-5}$. It can be seen from Figure 6a that ECC depends on both the number of users and the value of *j*. Specifically, ECC will increase with the number of the users for a large *j*, but will decrease for a small *j*. This is because the situation with more users means poorest channel condition is worse than that of the case with fewer users. Thus, ECC will decrease with the increasing of users when j=1. On the contrary, ECC is proportional to the number of users when j=N, since the best channel condition is better than that of the case with fewer users. In addition, the total collected energy is taken into account. Obviously, for any *j*, the larger the number of users, the higher the collected energy will be. Moreover, a smaller *j* means the user with lower N-SNR will be selected to transmit the data, and the users with better N-SNR will perform the energy collection, which will result in more total collected energy as shown in Figure 6b.

## 7. Conclusions

As an important component of modem communication systems, WSNs are used to collect, process and deliver information in particular locations by multi-hops. The whole network performance, however, is seriously affected by the cooperative work and the limited energy supply. Traditional sleeping mechanisms could reduce the network energy consumption partly, but cannot solve the problems of energy constraints fundamentally. Under this circumstance, synchronous wireless information and power transmission (SWIPT) system has been focused on in recent years, which can collect energy by radio frequency so as to provide long battery life for low-power devices. The problem of the balance between energy collection and information transmission is studied for SWIPT in this paper. Firstly, the information transmission model, the energy collecting model and the fading channel model are established, based on which IS2M, INS2M and IETSM are put forward. The users are sorted in ascending order according to SNR by IS2M. The balance between energy collection and data transmission can be achieved by selecting different ordinals. Considering that the users with poor channel environment may not be able to collect information in some special circumstances, normalized SNR (N-SNR) is adopted instead of SNR by a proposed modified mechanism known as INS2M to deal with the imbalance caused by low SNR users. IETSM is presented for the balanced information transmission for all users in the whole network. IETSM can adjust information transmission according to the average collected energy in order to improve the adaptability of the network. Simulations are performed comparing with the classical RR and ET mechanisms. The obtained results show that the proposed IS2M, INS2M and IETSM take both the energy collection and information transmission into account instead of best-effort strategy, so as to obtain a better performance of SWIPT by adjusting *j* or $S_{a}$. Moreover, INS2M and IETSM are able to provide long-term proportional fairness in information transmission and energy collection. Future research will focus on how to optimize the various scheduling mechanisms to be deployed in practical WSNs.

## Figures and Tables

**Table 1:** Variables and their meanings.

Variable	Meaning	Variable	Meaning
*n*	the ordinal of user	*j*	the ordinal of the selected user
*x*	the baseband symbol	*K*	shape parameter in Ricean Fading
η	the conversion efficiency	$\theta_{n}$	the phase of fading coefficient
$r_{n}$	the throughput of user *n*	*k*	shape parameter in Weibull Fading
*N*	the number of users	$\Omega_{n}$	the mean channel power gain of user *n*
β	a smoothing factor	$\sqrt{h_{n}}$	the amplitude of fading coefficient
$h_{n}$	the channel power gain	*a*,*b*	the smallest positive integers satisfying $\frac{b}{a}=k$
${\bar{\gamma }}_{n}$	the average SNR of user *n*	$Z_{n}$	zero-mean additive white Gaussian noise
*m*	a measure in Nakagami-m Fading	$p_{n}$	the channel access probability of user *n*

**Table 2:** PDF and CDF of the channel power gain in different fading models.

Channel Model	PDF $f_{h_{n}}\left(x\right)$	CDF$F_{h_{n}}\left(x\right)$	Parameters
Nakagami-*m*	$\frac{1}{\Gamma (m)}\lambda_{n}^{m}x^{m-1}e^{-\lambda_{n}x}$	$\begin{array}{c} 1-\frac{\Gamma (m,\lambda_{n}x)}{\Gamma (m)}\\ =1-e^{-\lambda_{n}x}\sum_{s=0}^{m-1}\frac{{\left(\lambda_{n}x\right)}^{s}}{s!} \end{array}$	$\lambda_{n}=\frac{m}{\Omega_{n}}$
Weibull	$k\lambda_{n}^{k}x^{k-1}e^{-{\left(\lambda_{n}x\right)}^{k}}$	$1-e^{-{\left(\lambda_{n}x\right)}^{k}}$	$\lambda_{n}=\frac{\Gamma \left(1+\frac{1}{k}\right)}{\Omega_{n}}$
Ricean	$\begin{array}{c} \frac{K+1}{\Omega_{n}}e^{-K-\frac{(K+1)x}{\Omega_{n}}}\\ I_{0}\left(2\sqrt{\frac{K(K+1)}{\Omega_{n}}x}\right) \end{array}$	$\begin{array}{c} 1-Q_{1}\left(\sqrt{2K},\sqrt{\frac{2(K+1)x}{\Omega_{n}}}\right)\\ \approx 1-e^{-\beta_{n}x^{\mu^{{}^{\prime }}}} \end{array}$	$\begin{array}{c} \beta_{n}=e^{\nu (\sqrt{2K})}{\sqrt{\frac{2(K+1)}{\Omega_{n}}}}^{\mu (\sqrt{2K})}\\ \mu^{{}^{\prime }}=\frac{\mu (\sqrt{2K})}{2} \end{array}$
Rayleigh	$\lambda_{n}e^{-\lambda_{n}x}$	$1-e^{-\lambda_{n}x}$	$\lambda_{n}=\frac{1}{\Omega_{n}}$

**Table 3:** The ergodic full-time access capacity of user *n* for different fading models.

Channel Model	$E[C_{U_{n,f}}]$
Nakagami-*m*	$\begin{array}{c} \frac{1}{\ln(2)\Gamma (m)}{\left(\frac{m}{{\bar{\gamma }}_{n}}\right)}^{m}G_{2,3}^{3,1}\left[\frac{m}{{\bar{\gamma }}_{n}}|\begin{array}{ccc} & -m, & 1-m,\\ 0, & -m, & -m, \end{array}\right] \end{array}$
Weibull	$\begin{array}{c} \frac{k\lambda_{n}^{\prime k}}{\ln(2)}\frac{{\sqrt{ab}}^{-1}}{{\left(2\pi \right)}^{\frac{a+2b-3}{2}}}G_{2b,a+2b}^{a+2b,b}\left[\frac{\lambda_{n}^{\prime ak}}{a^{a}}|\begin{array}{cc} ▵(b,-k), & ▵(b,1-k),\\ ▵(a,0), & ▵(b,-k),▵(b,-k) \end{array}\right] \end{array}$
Ricean	$\begin{array}{c} \frac{\left(1+K\right)e^{-K}}{\ln\left(2\right){\bar{\gamma }}_{n}}\sum_{i=0}^{\infty }\frac{1}{{\left(i!\right)}^{2}}{\left[\frac{K(1+K)}{{\bar{\gamma }}_{n}}\right]}^{i}G_{2,3}^{3,1}\left[\frac{\left(K+1\right)}{{\bar{\gamma }}_{n}}|\begin{array}{ccc} & -1-i, & -i,\\ 0, & -1-i, & -1-i, \end{array}\right] \end{array}$
Rayleigh	$\begin{array}{c} \frac{1}{\ln(2)}e^{\frac{1}{{\bar{\gamma }}_{n}}}E_{1}\left(\frac{1}{{\bar{\gamma }}_{n}}\right) \end{array}$

**Table 4:** ECC of single user obtained by IS2M.

Channel Model	$E[C_{j,U_{n}}]$
Nakagami-*m*	$\begin{array}{c} \frac{\lambda_{n}^{m}}{\ln(2)\Gamma (m)}\sum_{r=0}^{j-1}{\left(-1\right)}^{r}\sum_{U_{n,r}}\sum_{S_{m,r}}\frac{\prod_{t=1}^{N-j+r}\lambda_{u_{t}}^{s_{t}}}{\prod_{t=1}^{N-j+r}s_{t}!}{\left(\lambda_{n}+\sum_{t=1}^{N-j+r}\lambda_{u_{t}}\right)}^{-\left(m+\sum_{t=1}^{N-j+r}s_{t}\right)}\\ \Gamma \left(m+\sum_{t=1}^{N-j+r}s_{t}\right)\left(\Psi \left(m+\Sigma_{t=1}^{N-j+r}s_{t}\right)+\ln\left(\frac{\bar{\gamma }}{\lambda_{n}+\Sigma_{t=1}^{N-j+r}\lambda_{u_{t}}}\right)\right) \end{array}$
Weibull	$\begin{array}{c} \frac{1}{\ln(2)}\lambda_{n}^{k}\sum_{r=0}^{j-1}{\left(-1\right)}^{r}\sum_{U_{n,r}}{\left(\lambda_{n}^{k}+\sum_{t=1}^{N-j+r}\lambda_{u_{t}}^{k}\right)}^{-1}\left(\ln\left(\bar{\gamma }\right)-\frac{1}{k}\left(\ln\left(\lambda_{n}^{k}+\sum_{t=1}^{N-j+r}\lambda_{u_{t}}^{k}\right)+C\right)\right) \end{array}$
Ricean	$\begin{array}{c} \frac{1}{\ln(2)}\beta_{n}\sum_{r=0}^{j-1}{\left(-1\right)}^{r}\sum_{U_{n,r}}{\left(\beta_{n}+\sum_{t=1}^{N-j+r}\beta_{u_{t}}\right)}^{-1}\left(\ln\left(\bar{\gamma }\right)-\frac{1}{\mu^{{}^{\prime }}}\left(\ln\left(\beta_{n}+\sum_{t=1}^{N-j+r}\beta_{u_{t}}\right)+C\right)\right) \end{array}$
Rayleigh	$\begin{array}{c} \frac{1}{\ln(2)}\lambda_{n}\sum_{r=0}^{j-1}{\left(-1\right)}^{r}\sum_{U_{n,r}}{\left(\lambda_{n}+\sum_{t=1}^{N-j+r}\lambda_{u_{t}}\right)}^{-1}e^{\frac{1}{\bar{\gamma }}\left(\lambda_{n}+\sum_{t=1}^{N-j+r}\lambda_{u_{t}}\right)}E_{1}\left(\frac{1}{\bar{\gamma }}\left(\lambda_{n}+\sum_{t=1}^{N-j+r}\lambda_{u_{t}}\right)\right) \end{array}$

**Table 5:** ACE of single user obtained by IS2M.

Channel Model	$E[{\mathrm{EC}}_{j,U_{n}}]$
Nakagami-*m*	$\begin{array}{c} \eta P\Omega_{n}-\eta P\frac{\lambda_{n}^{m}}{\Gamma (m)}\sum_{r=0}^{j-1}{\left(-1\right)}^{r}\sum_{U_{n,r}}\sum_{S_{m,r}}\frac{\prod_{t=1}^{N-j+r}\lambda_{u_{t}}^{s_{t}}}{\prod_{t=1}^{N-j+r}s_{t}!}{\left(\lambda_{n}+\sum_{t=1}^{N-j+r}\lambda_{u_{t}}\right)}^{-\left(m+1+\sum_{t=1}^{N-j+r}s_{t}\right)}\\ \Gamma \left(m+1+\sum_{t=1}^{N-j+r}s_{t}\right) \end{array}$
Weibull	$\begin{array}{c} \eta P\left(\Omega_{n}-\lambda_{n}^{k}\Gamma \left(1+\frac{1}{k}\right)\sum_{r=0}^{j-1}{\left(-1\right)}^{r}\sum_{U_{n,r}}{\left(\lambda_{n}^{k}+\sum_{t=1}^{N-j+r}\lambda_{u_{t}}^{k}\right)}^{-\left(1+\frac{1}{k}\right)}\right) \end{array}$
Ricean	$\begin{array}{c} \eta P\left(\Omega_{n}-\beta_{n}\Gamma \left(1+\frac{1}{\mu^{{}^{\prime }}}\right)\sum_{r=0}^{j-1}{\left(-1\right)}^{r}\sum_{U_{n,r}}{\left(\beta_{n}^{+}\sum_{t=1}^{N-j+r}\beta_{u_{t}}\right)}^{-\left(1+\frac{1}{\mu^{{}^{\prime }}}\right)}\right) \end{array}$
Rayleigh	$\begin{array}{c} \eta P\left(\Omega_{n}-\lambda_{n}\sum_{r=0}^{j-1}{\left(-1\right)}^{r}\sum_{U_{n,r}}{\left(\lambda_{n}+\sum_{t=1}^{N-j+r}\lambda_{u_{t}}\right)}^{-2}\right) \end{array}$

**Table 6:** ECC of a single user obtained by INS2M.

Channel Model	$E[C_{j,U_{n}}]$
Nakagami-*m*	$\begin{array}{c} \frac{1}{\ln(2)\Gamma (m)}\left(\begin{array}{c} N-1\\ j-1 \end{array}\right)\sum_{l=N-j}^{N-1}{\left(-1\right)}^{l-N+j}\left(\begin{array}{c} j-1\\ N-l-1 \end{array}\right)\frac{l!}{{\left(1+l\right)}^{m}}\\ \sum_{L_{m,l}}\left(\prod_{s=0}^{m-1}\frac{{\left(\frac{1}{s!{\left(1+l\right)}^{s}}\right)}^{i_{s}}}{i_{s}!}\right)\Gamma \left(m+\sum_{s=0}^{m-1}si_{s}\right)\left(\Psi \left(m+\Sigma_{s=0}^{m-1}si_{s}\right)+\ln\left(\frac{{\bar{\gamma }}_{n}}{m(1+l)}\right)\right) \end{array}$
Weibull	$\begin{array}{c} \frac{1}{\ln(2)}\left(\begin{array}{c} N-1\\ j-1 \end{array}\right)\sum_{l=0}^{j-1}\frac{{\left(-1\right)}^{l}\left(\begin{array}{c} j-1\\ l \end{array}\right)}{N-j+l+1}\\ \left(\ln\left({\bar{\gamma }}_{n}\right)-\frac{1}{k}\left(\ln\left(\left(N-j+l+1\right)\Gamma {\left(1+\frac{1}{k}\right)}^{k}\right)+C\right)\right) \end{array}$
Ricean	$\begin{array}{c} \frac{1}{\ln(2)}\left(\begin{array}{c} N-1\\ j-1 \end{array}\right)\sum_{l=0}^{j-1}\frac{{\left(-1\right)}^{l}\left(\begin{array}{c} j-1\\ l \end{array}\right)}{N-j+l+1}\left(\ln\left({\bar{\gamma }}_{n}\right)-\frac{1}{\mu^{{}^{\prime }}}\left(\ln\left((N-j+l+1)\beta \right)+C\right)\right) \end{array}$
Rayleigh	$\begin{array}{c} \frac{1}{\ln(2)}\left(\begin{array}{c} N-1\\ j-1 \end{array}\right)\sum_{l=0}^{j-1}\frac{{\left(-1\right)}^{l}\left(\begin{array}{c} j-1\\ l \end{array}\right)}{N-j+l+1}e^{\frac{\left(N-j+l+1\right)}{{\bar{\gamma }}_{n}}}E_{1}\left(\frac{\left(N-j+l+1\right)}{{\bar{\gamma }}_{n}}\right) \end{array}$

**Table 7:** ACE of a single user obtained by INS2M.

Channel Model	$E[{\mathrm{EC}}_{j,U_{n}}]$
Nakagami-*m*	$\begin{array}{c} \eta P\Omega_{n}-\eta P\Omega_{n}\frac{1}{\Gamma (m+1)}\left(\begin{array}{c} N-1\\ j-1 \end{array}\right)\sum_{l=N-j}^{N-1}{\left(-1\right)}^{l-N+j}\left(\begin{array}{c} j-1\\ N-l-1 \end{array}\right)\frac{l!}{{\left(1+l\right)}^{m+1}}\\ \sum_{L_{m,l}}\left(\prod_{s=0}^{m-1}\frac{{\left(\frac{1}{s!{\left(1+l\right)}^{s}}\right)}^{i_{s}}}{i_{s}!}\right)\Gamma \left(m+1+\sum_{s=0}^{m-1}si_{s}\right) \end{array}$
Weibull	$\begin{array}{c} \eta P\Omega_{n}\left(1-\left(\begin{array}{c} N-1\\ j-1 \end{array}\right)\sum_{l=0}^{j-1}{\left(-1\right)}^{l}\left(\begin{array}{c} j-1\\ l \end{array}\right){\left(N-j+l+1\right)}^{-\left(1+\frac{1}{k}\right)}\right) \end{array}$
Ricean	$\begin{array}{c} \eta P\Omega_{n}\left(1-\left(\begin{array}{c} N-1\\ j-1 \end{array}\right)\sum_{l=0}^{j-1}{\left(-1\right)}^{l}\left(\begin{array}{c} j-1\\ l \end{array}\right){\left(N-j+l+1\right)}^{-\left(1+\frac{1}{\mu^{{}^{\prime }}}\right)}\right) \end{array}$
Rayleigh	$\begin{array}{c} \eta P\Omega_{n}\left(1-\frac{1}{N}\sum_{l=N-j+1}^{n}\frac{1}{l}\right) \end{array}$

## Abbreviations

The following abbreviations are used in this manuscript:

WSNs	Wireless Sensor Networks
SNR	Signal Noise Ratio
TDMA	Time Division Multiple Access
IS2M	Improved SNR Scheduling Mechanism
INS2M	Improved N-SNR Scheduling Mechanism
IETSM	Improved Equal Throughput Scheduling Mechanism
EC	Energy Collecting
RF	Radio Frequency
SWIPT	Synchronous Wireless Information and Power Transmission
R-E	Rate-Energy
MD	Message Decoding
MUD	Multi-User Diversity
ACE	Average Collected Energy
ECC	Ergodic Channel Capacity
NAP	Network Access Port
STs	Subscriber Terminals
RR	Round Robin
ET	Equal Throughout
CAP	Channel Access Probability
PDF	Probability Density Function
CDF	Cumulative Distribution Function
